# Effectiveness of Bariatric Surgery for Improving Thyroid Function and Reducing Levothyroxine Dose in Patients With Obesity and Overt Hypothyroidism

**DOI:** 10.7759/cureus.38780

**Published:** 2023-05-09

**Authors:** Hanan H Alfaifi, Manar A Altowairgi, Rawan G Algethami, Atheer H Altowairqi, Hajar D Althomali, Owaid M Almalki

**Affiliations:** 1 Medicine and Surgery, College of Medicine, Taif University, Taif, SAU; 2 Department of Surgery, College of Medicine, Taif University, Taif, SAU

**Keywords:** sleeve gastrectomy, levo-thyroxine treatment, hypothyroidism, obesity, bariatric surgeries

## Abstract

Background: Hypothyroidism is a major hormonal condition that affects more women than men in Saudi Arabia. Studies indicate a bidirectional link between hypothyroidism and obesity, which may improve following bariatric surgery (BS). The focus of this research is to assess how hypothyroidism patients' thyroid function and levothyroxine dosage are impacted by bariatric surgery.

Methodology: This was an observational retrospective study conducted in two centres at Taif, Saudi Arabia. All morbidly obese patients who were diagnosed with overt hypothyroidism and underwent laparoscopic sleeve gastrectomy from January 2016 to December 2021 were included. The changes in the thyroid profile and the changes in the doses or cessation of levothyroxine were evaluated after the laparoscopic sleeve gastrectomy.

Results: Our results demonstrate that a total of 70 patients dominated by women out of 1202 from both centers who meet our inclusion criteria showed a statistically significant decrease on comparison of clinical parameters (thyroid-stimulating hormone [TSH], free T4 [FT4], free T3 [FT3], levothyroxine [L-T4]) before and after BS. The average TSH levels were determined to be 4.45 ± 4.41 mIU/L prior to BS, and they significantly decreased (3.17 ± 2.77 mIU/L) following BS (p=0.009). When compared to before BS (13.17 ± 2.73 pmol/L), the mean FT4 levels after BS (11.63 ± 5.88 pmol/L) exhibited a significant decline (p=0.046). The mean FT3 levels before and after BS also were statistically significantly lower (1.94 ± 2.12 pg/mL) than before (2.75 ± 1.96 pg/mL), p=0.009. The mean L-T4 levels after BS considerably decreased from before BS (98.68 ± 56.18 mcg) to after BS (79.39 ± 41.49 mcg), p=0.046.

Conclusion: Better thyroid profiles and lower levothyroxine dosage show that bariatric surgery improves hypothyroidism.

## Introduction

The thyroid gland is a highly vascular endocrine gland responsible for several vital processes in the human body, including metabolism, growth, and development, which are regulated by triiodothyronine (T3) and thyroxine (T4) [1]. Primary overt hypothyroidism is characterized by insufficient synthesis of T3 and T4, accompanied by an increased serum thyroid stimulating hormone (TSH) level [2]. Hypothyroidism can affect the functionality of several organs and tissues, and its signs and symptoms range from minor to life-threatening, depending on the severity of the hormone deficit. Early detection of hypothyroidism can be achieved through a thyroid function test that evaluates hormone levels. The only medication proven to effectively treat primary overt hypothyroidism is levothyroxine (L-T4) [3].

Hypothyroidism is more commonly observed in Saudi women than men, consistent with its higher global prevalence among women. A recent hospital-based study conducted in Jeddah, Saudi Arabia, reported a hypothyroidism prevalence of 29.1% among 3,872 participants, the majority (85.7%) of whom were female [4-6]. Obesity is a significant threat to public health, as it is a leading cause of many life-threatening diseases and conditions including hormonal abnormalities in the thyroid glands [7]. There is strong evidence of a bidirectional relationship between obesity and thyroid dysfunction [8]. Weight gain is a common symptom of hypothyroidism, which accordingly has a relatively high incidence among individuals with obesity. Thyroid hormones play a role in regulating the metabolic rate. A systematic review and meta-analysis conducted in 2019 confirmed that obesity is a risk factor for overt hypothyroidism [9,10]. Furthermore, several studies have indicated that individuals with obesity have a 70% increased risk of developing subclinical hypothyroidism [8,11].

Bariatric surgery (BS) is a weight-reduction surgery that involves multiple procedures designed to restrict the ability of the body to ingest and absorb food, by decreasing stomach capacity or intestinal length [12]. In 2016, Al-Khaldi [13] reported that approximately 15,000 BS are performed annually in Saudi Arabia. The three main types of BS are restrictive, malabsorptive, and both in combination [14]. Restrictive surgery involves removing and narrowing the stomach with laparoscopic sleeve gastrectomy (LSG); this accounts for half of all bariatric procedures performed globally [15]. Laparoscopic Roux-en-Y gastric bypass is a combination of restrictive and malabsorptive surgery that accounts for 30% of all bariatric procedures. Additionally, less frequently performed procedures included duodenal switch (DS), single-anastomosis duodeno-ileal bypass (SADI), and one-anastomosis gastric bypass (OAGB) [14,15]. The Saudi Arabian Society for Metabolic and Bariatric Surgery has established inclusion criteria for BS, which include an age of 14-65 years, body mass index (BMI) of ≥ 35 kg/m2 (or ≥ 30 kg/m2 if there are associated comorbidities), and failure to achieve weight loss through nonsurgical methods [16]. Several studies have demonstrated improvements in the thyroid profile following BS in obese patients [17-19]. 

Few studies have evaluated the effect of BS on hypothyroidism in Saudi Arabia, and no studies have been conducted in the Taif province. This study aimed to evaluate the ability of BS to improve overt hypothyroidism.

## Materials and methods

A retrospective observational study was conducted at two main hospitals in Taif, Saudi Arabia. Data of patients who underwent LSG or Roux-en-Y gastric bypass and were diagnosed with hypothyroidism between 2016 and 2021 were extracted from the hospital database. In total, 70 of 1,202 patients met the study inclusion criteria. The patients were required to have a body mass index (BMI) ≥ 40 kg/m2 (or ≥ 35 kg/m2 if associated with comorbidities such as hypertension, diabetes, hyperlipidemia, and obstructive sleep apnea). Hypothyroidism was diagnosed by an endocrinologist or family physician during two or more preoperative visits. The patients had no history of other thyroid diseases, such as tumors or surgery. Patients who were lost to follow-up were excluded from the study. 

The preoperative data extracted from the medical records were baseline demographic information, BMI, L-T4 doses, and serum levels of TSH, free T4 (FT4), and free T3 (FT3). Overt hypothyroidism was diagnosed in patients who received L-T4 treatment and had elevated TSH and reduced T4 levels. Subclinical hypothyroidism was diagnosed in patients who had not received L-T4, and had elevated TSH and normal T4 levels. The final postoperative visits to surgery, endocrinology, and family medicine clinics, as well as the thyroid hormone levels and L-T4 doses, were recorded. The normalization of thyroid hormone levels after L-T4 administration indicated resolution of overt hypothyroidism, whereas a decrease in the L-T4 dose was considered to denote improvement, and no dose change indicated no improvement. 

Statistical analysis was performed using SPSS software (version 23.0; IBM Corp., Armonk, NY, USA). Descriptive analysis was performed for categorical data, and the results are presented as frequencies and percentages. Tables and figures summarizing the data are presented. The Shapiro-Wilk test was used to determine the normality of the data distribution. Mean and standard deviation values were calculated for normally distributed data, whereas the median and interquartile range were calculated for non-normally distributed data. Continuous and categorical variables were compared using Student’s t-test or the Mann-Whitney U test, as appropriate. P-values < 0.05 were considered statistically significant. The study protocol was approved by the institutional review boards of both hospitals.

## Results

We evaluated the effect of BS on thyroid function by analyzing clinical parameters, including L-T4 doses and serum levels of TSH, FT4, and FT3. The analysis was based on the availability of data on each clinical parameter in the patients’ medical records. The mean TSH level before BS was 4.45 ± 4.41 mIU/L, which was significantly reduced after BS to 3.17 ± 2.77 mIU/L (p = 0.009). The mean FT4 level after BS (11.63 ± 5.88 pmol/L) was significantly lower than before BS (13.17 ± 2.73 pmol/L) (p = 0.046). A statistically significant reduction was observed in the mean FT3 level, from 2.75 ± 1.96 pg/mL before BS to 1.94 ± 2.12 pg/mL thereafter (p = 0.009). Moreover, the mean L-T4 dose administered after BS was significantly lower (79.39 ± 41.49 mcg) than that before BS (98.68 ± 56.18 mcg) (p = 0.046) (Table 1).

**Table 1:** Comparison of clinical parameters before and after TSH: thyroid-stimulating hormone, FT4: free T4, FT3, free T3, L-T4: levothyroxine

		N(%)	Mean	Std. Deviation	p value*
TSH	Pre TSH	66(94)	4.45	4.41	0.009
	Post TSH	66(94)	3.17	2.77	
FT4	Pre FT4	62(89)	13.17	2.73	0.046
	Post FT4	62(89)	11.63	5.88	
FT3	Pre FT3	35(50)	2.75	1.96	0.009
	Post FT3	35(50)	1.94	2.12	
L-T4 (mcg)	Pre L-T4	57(81)	98.68	56.18	0.046
	Post L-T4	57(81)	79.39	41.49	

When comparing the mean TSH level before and after BS separately in females and males, we found no statistically significant differences (p = 0.056 and p = 0.065, respectively). Similarly, the mean FT4 level did not show statistically significant differences after BS in females (p = 0.066) or males (p = 0.323). However, we observed a significant reduction in the mean FT3 level after BS in females (p = 0.014), but not in males (p = 0.500). In addition, a statistically significant reduction in the mean L-T4 dose administered after BS was observed in females (p = 0.001) compared to males (p = 0.0197), as shown in Table 2.

**Table 2:** Comparison of clinical parameters before and after based on gender TSH: thyroid-stimulating hormone, FT4: free T4, FT3, free T3, L-T4: levothyroxine

Gender			N(%)	Mean	SD	P value*
Female	TSH	Pre TSH	59(84)	3.85	2.80	0.056
		Post TSH	59(84)	2.97	2.30	
	FT4	Pre FT4	55(79)	13.12	2.73	0.066
		Post FT4	55(79)	11.55	6.20	
	FT3	Pre FT3	33(47)	2.78	1.93	0.014
		Post FT3	33(47)	2.05	2.13	
	L-T4 mcg	Pre L-T4 mcg	50(71)	94.50	56.76	0.001
		Post L-T4 mcg	50(71)	76.00	42.55	
Male	TSH	Pre TSH	7(10)	9.46	10.11	0.065
		Post TSH	7(10)	4.86	5.32	
	FT4	Pre FT4	7(10)	13.61	2.90	0.323
		Post FT4	7(10)	12.32	2.29	
	FT3	Pre FT3	2(3)	2.31	3.27	0.500
		Post FT3	2(3)	0.00	0.00	
	L-T4 mcg	Pre L-T4 mcg	7(10)	128.57	44.32	0.197
		Post L-T4 mcg	7(10)	103.57	22.49	

Table 3 compares thyroid hormones among age groups. The mean TSH level before and after BS showed statistically significant differences only in the 46-55 years age group (p = 0.012). Furthermore, we compared the mean FT4 and FT3 levels, as well as the mean L-T4 dose administered in the 36-45 years age group, and observed statistically significant differences in comparison to the other age groups (p < 0.05).

**Table 3:** Comparison of clinical parameters before and after based on age TSH: thyroid-stimulating hormone, FT4: free T4, FT3, free T3, L-T4: levothyroxine

			Age (years)				
			<=25	26-35	36-45	46-55	>=56
TSH	Pre TSH	N(%)	5(7)	12(17)	21(30)	24(34)	4(6)
		Mean	2.31	3.18	4.00	6.39	1.58
		SD	1.02	2.25	2.43	6.29	1.69
	Post TSH	N(%)	5(7)	12(17)	21(30)	24(34)	4(6)
		Mean	2.53	3.36	2.80	3.93	0.84
		SD	1.85	2.78	2.16	3.38	0.99
	p value*		0.862	0.889	0.092	0.012	0.279
FT4	Pre FT4	N(%)	5(7)	12(17)	19(27)	23(33)	3(4)
		Mean	13.13	13.53	13.35	12.77	13.75
		SD	1.60	2.60	1.80	3.59	3.31
	Post FT4	N(%)	5(7)	12(17)	19(27)	23(33)	3(4)
		Mean	10.55	12.83	10.28	12.79	8.38
		SD	6.16	5.40	5.16	6.50	7.26
	p value*		0.450	0.639	0.013	0.987	0.420
FT3	Pre FT3	N(%)	3(4)	10(14)	9(13)	11(16)	2(3)
		Mean	3.96	3.39	3.03	2.10	0.00
		SD	1.04	1.89	1.79	2.05	0.00
	Post FT3	N(%)	3(4)	10(14)	9(13)	11(16)	2(3)
		Mean	2.87	3.11	1.55	1.28	0.00
		SD	2.55	2.36	1.84	1.81	0.00
	p value*		0.424	0.586	0.041	0.163	—
L-T4 mcg	Pre L-T4 mcg	N(%)	5(7)	7(10)	18(26)	23(33)	4(6)
		Mean	55.00	71.43	118.06	98.91	112.50
		SD	37.08	50.89	65.20	49.70	47.87
	Post L-T4 mcg	N(%)	5(7)	7(10)	18(26)	23(33)	4(6)
		Mean	50.00	60.71	87.50	84.78	81.25
		SD	30.62	57.48	42.23	38.24	23.94
	p value*		0.838	0.078	0.002	0.102	0.141
* p value<0.05 is considered statistically significant							

## Discussion

The available evidence supports the effectiveness of BS for treating obesity-related comorbidities [20-22]. Our study has demonstrated that BS can improve thyroid function, as indicated by the significant reductions seen in L-T4 doses and serum levels of TSH, FT4, and FT3. Several studies have demonstrated that BS-induced weight loss reduces TSH levels in euthyroid and hypothyroid patients [23,24]. In addition, BS affects the pharmacokinetics and bioavailability of drugs. Optimal T4 absorption requires an acidic stomach environment [25]. In 2020, Khan et al. [26] reported a significant reduction in thyroid replacement dose following BS among hypothyroidism patients. Although several comparative studies have been conducted, none have established the superiority of one surgical method over another in terms of improving thyroid function. It has been demonstrated that restrictive and bypass surgeries significantly reduce the thyrotropin-releasing hormone dose required [24,27-30]. LSG is a reliable first-line surgical option for super obesity and metabolic disorders. Furthermore, one-anastomosis gastric bypass (OAGB) has a similar surgical risk to LSG, but results in greater weight reduction [31]. 

Our findings suggest that BS alters TSH levels; however, further studies are needed to verify our results. Previous studies have provided contradictory findings regarding the association between TSH level and weight loss [32-36]. Although the present and previous studies have shown an association between thyroid hormone replacement and BS outcomes, the underlying mechanism is unclear. In adults with euthyroid obesity, a reduction in BMI indicates improved thyroid function. Moreover, surgical weight reduction has been associated with adipokine release, which affects thyroid hormone levels [23,37]. Leptin, a hormone secreted by adipose tissue, regulates the expression of the thyrotropin-releasing hormone gene [38]. A reduction in leptin level has been observed following weight loss induced by LSG or laparoscopic Roux-en-Y gastric bypass [39]. Weight loss following LSG is associated with a significant reduction in leptin levels, potentially leading to a decreased TSH level. LSG induces alterations in peptide hormone profiles, particularly those of pancreatic and intestinal origin, which are implicated in the metabolic benefits of BS [40,41]. In addition, BS induces alterations in serum FT3, FT4, and TSH levels by reducing adipose tissue. Studies have shown that obese individuals exhibit reduced expression of thyroid hormone receptor genes, particularly the TSH receptor, in both subcutaneous and visceral adipose tissue [42]. The primary mechanism through which adipokines, particularly leptin, affect thyroid hormone levels is widely accepted. Leptin, secreted by adipose tissue, stimulates thyroid function by elevating TSH and FT3 levels. Therefore, decreased leptin levels following surgery-induced weight loss and adipose tissue reduction can result in a low TSH level [43,44]. Treatment of morbid obesity can reduce elevations in the TSH level caused by the overactivation of the thyroid axis, in turn due to various pathophysiological processes and endocrine adaptations [45]. Studies have reported that ghrelin levels decrease following LSG and Roux-en-Y gastric bypass, whereas they remain unchanged after laparoscopic adjustable gastric banding [46]. Removal of the gastric fundus during surgery reduces ghrelin levels, which can affect TSH levels. Moreover, lowering ghrelin levels can also help reduce TSH levels, where weight loss triggers changes in hormone levels [47]. 

Our study had some limitations. First, thyroid function was evaluated by measuring L-T4 doses and serum TSH, FT4, and FT3 levels six months after surgery. Re-evaluating the same measures after 12 and 24 months, or longer, would have provided stronger evidence. TSH levels can be influenced by variables other than weight reduction following BS, despite the application of exclusion criteria, including drug use and thyroid disorders. For example, seasonal variations can lead to fluctuations in TSH levels, which decrease in summer and increase in winter [48]. Therefore, longitudinal studies with a larger sample are needed to confirm our findings. In addition, BMI was not recorded in all follow-up visits, which limited the valuation of the association between weight loss and thyroid profile improvement.

Our study had certain advantages as well. Unlike previous studies that only measured TSH levels, we evaluated L-T4 doses, as well as serum FT4 and FT3 levels. Our study emphasizes that morbidly obese individuals frequently exhibit a return to normal TSH levels following BS. Therefore, TSH values in these individuals should be interpreted with caution. Our results contribute to the growing knowledge of how obesity impacts thyroid hormones. Further research is needed to determine the factors driving low TSH levels after BS.

## Conclusions

In conclusion, the results of our research indicate that bariatric surgery generally has a beneficial effect on patients suffering from hypothyroidism. Signs of this include an improved thyroid profile as well as a decreased amount of L-T4 dosage. The correlation between body mass index and thyroid hormones is further strengthened by this finding. This suggests that weight loss can have a positive impact on thyroid function and may reduce the need for medication in some cases.

## References

[1] Armstrong M, Asuka E, Fingeret A (2023). Physiology, Thyroid Function. https://www.ncbi.nlm.nih.gov/books/NBK537039/.

[2] Sheehan MT (2016). Biochemical testing of the thyroid: TSH is the best and, oftentimes, only test needed-a review for primary care. Clin Med Res.

[3] (2016). Thyroid Disorders: Basic Science and Clinical Practice.

[4] Aljabri KS, Alnasser IM, Facharatz BS, Bokhari SA, Alshareef MA, Khan PM (2019). The frequency of hypothyroidism in Saudi community-based hospital: a retrospective single centre study. Trends Diabetes Metab.

[5] Alruwaili AM, Alshalan MF, Alfuhigi ZM (2018). Prevalence of hypothyroidism and its associated risk factors in Arar City, Saudi Arabia. Egypt J Hosp Med.

[6] Hasanato R, Mirah JA, Al-Shahrani N, Alfulayyih N, Almutairi A, Ogailan B, Fatma S (2017). Incidence of thyroid diseases in female Saudi adults visiting a tertiary care hospital in Riyadh. Epidemiology.

[7] Pedro J, Cunha F, Souteiro P (2018). The effect of the bariatric surgery type on the levothyroxine dose of morbidly obese hypothyroid patients. Obes Surg.

[8] Sanyal D, Raychaudhuri M (2016). Hypothyroidism and obesity: an intriguing link. Indian J Endocrinol Metab.

[9] Almunif DS, Bamehriz F, Althuwaini S, Almigbal TH, Batais MA (2020). The effect of laparoscopic sleeve gastrectomy on serum thyroid-stimulating hormone levels in obese patients with overt and subclinical hypothyroidism: a 7-year retrospective study. Obes Surg.

[10] Song RH, Wang B, Yao QM, Li Q, Jia X, Zhang JA (2019). The impact of obesity on thyroid autoimmunity and dysfunction: a systematic review and meta-analysis. Front Immunol.

[11] Yan Y, Xu M, Wu M (2022). Obesity is associated with subclinical hypothyroidism in the presence of thyroid autoantibodies: a cross-sectional study. BMC Endocr Disord.

[12] Gilmartin J, Bath-Hextall F, Maclean J, Stanton W, Soldin M (2016). Quality of life among adults following bariatric and body contouring surgery: a systematic review. JBI Database System Rev Implement Rep.

[13] Al-Khaldi Y (20161). Bariatric surgery in Saudi Arabia: the urgent need for standards. Saudi J Obes.

[14] Khwaja HA, Bonanomi G (2010). Bariatric surgery: techniques, outcomes and complications. Curr Anaesth Crit Care.

[15] Felsenreich DM, Bichler C, Langer FB, Gachabayov M, Prager G (2020). Sleeve gastrectomy: surgical technique, outcomes, and complications. Surg Technol Int.

[16] Aljaaly EA (2021). Perioperative nutrition care and dietetic practices in the scope of bariatric surgery in Saudi Arabia using adapted protocols for evaluation. SAGE Open Med.

[17] Bian N, Sun X, Zhou B (2021). Obese patients with higher TSH levels had an obvious metabolic improvement after bariatric surgery. Endocr Connect.

[18] Jabbour G, Salman A (2021). Bariatric surgery in adults with obesity: the impact on performance, metabolism, and health indices. Obes Surg.

[19] Juiz-Valiña P, Outeiriño-Blanco E, Pértega S (2019). Effect of weight loss after bariatric surgery on thyroid-stimulating hormone levels in euthyroid patients with morbid obesity. Nutrients.

[20] Courcoulas AP, Christian NJ, Belle SH (2013). Weight change and health outcomes at 3 years after bariatric surgery among individuals with severe obesity. JAMA.

[21] Nguyen NT, Varela JE, Sabio A, Naim J, Stamos M, Wilson SE (2006). Reduction in prescription medication costs after laparoscopic gastric bypass. Am Surg.

[22] Perry CD, Hutter MM, Smith DB, Newhouse JP, McNeil BJ (2008). Survival and changes in comorbidities after bariatric surgery. Ann Surg.

[23] Abu-Ghanem Y, Inbar R, Tyomkin V, Kent I, Berkovich L, Ghinea R, Avital S (2015). Effect of sleeve gastrectomy on thyroid hormone levels. Obes Surg.

[24] Raftopoulos Y, Gagné DJ, Papasavas P, Hayetian F, Maurer J, Bononi P, Caushaj PF (2004). Improvement of hypothyroidism after laparoscopic Roux-en-Y gastric bypass for morbid obesity. Obes Surg.

[25] Padwal R, Brocks D, Sharma AM (2010). A systematic review of drug absorption following bariatric surgery and its theoretical implications. Obes Rev.

[26] Khan WF, Singla V, Aggarwal S, Gupta Y (2020). Outcome of bariatric surgery on hypothyroidism: experience from a tertiary care center in India. Surg Obes Relat Dis.

[27] Aggarwal S, Modi S, Jose T (2014). Laparoscopic sleeve gastrectomy leads to reduction in thyroxine requirement in morbidly obese patients with hypothyroidism. World J Surg.

[28] Fazylov R, Soto E, Cohen S, Merola S (2008). Laparoscopic Roux-en-Y gastric bypass surgery on morbidly obese patients with hypothyroidism. Obes Surg.

[29] Fierabracci P, Martinelli S, Tamberi A (2016). Weight loss and variation of levothyroxine requirements in hypothyroid obese patients after bariatric surgery. Thyroid.

[30] Garg H, Priyadarshini P, Aggarwal S, Agarwal S, Chaudhary R (2017). Comparative study of outcomes following laparoscopic Roux-en-Y gastric bypass and sleeve gastrectomy in morbidly obese patients: a case control study. World J Gastrointest Endosc.

[31] Soong TC, Lee MH, Lee WJ, Almalki OM, Chen JC, Wu CC, Chen SC (2021). Long-term efficacy of bariatric surgery for the treatment of super-obesity: comparison of SG, RYGB, and OAGB. Obes Surg.

[32] Guan B, Chen Y, Yang J, Yang W, Wang C (2017). Effect of bariatric surgery on thyroid function in obese patients: a systematic review and meta-analysis. Obes Surg.

[33] Liu G, Liang L, Bray GA (2017). Thyroid hormones and changes in body weight and metabolic parameters in response to weight loss diets: the POUNDS LOST trial. Int J Obes (Lond).

[34] Neves JS, Castro Oliveira S, Souteiro P (2018). Effect of weight loss after bariatric surgery on thyroid-stimulating hormone levels in patients with morbid obesity and normal thyroid function. Obes Surg.

[35] Yang J, Gao Z, Yang W, Zhou X, Lee S, Wang C (2017). Effect of sleeve gastrectomy on thyroid function in chinese euthyroid obese patients. Surg Laparosc Endosc Percutan Tech.

[36] Zhang H, Liu W, Han X, Yu H, Zhang P, Jia W (2017). Effect of laparoscopic Roux-en-Y gastric bypass surgery on thyroid hormone levels in Chinese patients, could it be a risk for thyroid nodules?. Obes Surg.

[37] Chikunguwo S, Brethauer S, Nirujogi V, Pitt T, Udomsawaengsup S, Chand B, Schauer P (2007). Influence of obesity and surgical weight loss on thyroid hormone levels. Surg Obes Relat Dis.

[38] Guo F, Bakal K, Minokoshi Y, Hollenberg AN (2004). Leptin signaling targets the thyrotropin-releasing hormone gene promoter in vivo. Endocrinology.

[39] Terra X, Auguet T, Guiu-Jurado E (2013). Long-term changes in leptin, chemerin and ghrelin levels following different bariatric surgery procedures: Roux-en-Y gastric bypass and sleeve gastrectomy. Obes Surg.

[40] Ramón JM, Salvans S, Crous X (2012). Effect of Roux-en-Y gastric bypass vs sleeve gastrectomy on glucose and gut hormones: a prospective randomised trial. J Gastrointest Surg.

[41] Meek CL, Lewis HB, Reimann F, Gribble FM, Park AJ (2016). The effect of bariatric surgery on gastrointestinal and pancreatic peptide hormones. Peptides.

[42] Nannipieri M, Cecchetti F, Anselmino M (2009). Expression of thyrotropin and thyroid hormone receptors in adipose tissue of patients with morbid obesity and/or type 2 diabetes: effects of weight loss. Int J Obes (Lond).

[43] Kozłowska L, Rosołowska-Huszcz D (2004). Leptin, thyrotropin, and thyroid hormones in obese/overweight women before and after two levels of energy deficit. Endocrine.

[44] Rosenbaum M, Leibel RL (2014). 20 years of leptin: role of leptin in energy homeostasis in humans. J Endocrinol.

[45] Park HK, Ahima RS (2015). Physiology of leptin: energy homeostasis, neuroendocrine function and metabolism. Metabolism.

[46] Langer FB, Reza Hoda MA, Bohdjalian A (2005). Sleeve gastrectomy and gastric banding: effects on plasma ghrelin levels. Obes Surg.

[47] Emami A, Nazem R, Hedayati M (2014). Is association between thyroid hormones and gut peptides, ghrelin and obestatin, able to suggest new regulatory relation between the HPT axis and gut?. Regul Pept.

[48] Yoshihara A, Noh JY, Watanabe N (2018). Seasonal changes in serum thyrotropin concentrations observed from big data obtained during six consecutive years from 2010 to 2015 at a single hospital in Japan. Thyroid.

